# Spatial topologies affect local food web structure and diversity in evolutionary metacommunities

**DOI:** 10.1038/s41598-017-01921-y

**Published:** 2017-05-12

**Authors:** Lev Bolchoun, Barbara Drossel, Korinna Theresa Allhoff

**Affiliations:** 1 Institute of Condensed Matter Physics, Technische Universität Darmstadt, Darmstadt, Germany; 2 Institute of Ecology and Environmental Sciences, Université Pierre et Marie Curie, Paris, France

## Abstract

An important challenge in theoretical ecology is to better predict ecological responses to environmental change, and in particular to spatial changes such as habitat fragmentation. Classical food-web models have focused on purely ecological predictions, without taking adaptation or evolution of species traits into account. We address this issue using an eco-evolutionary model, which is based on body masses and diets as the key traits that determine metabolic rates and trophic interactions. The model implements evolution by the introduction of new morphs that are related to the existing ones, so that the network structure itself evolves in a self-organized manner. We consider the coupling and decoupling of habitats in multi-trophic metacommunities consisting of 2 or 4 habitats. Our model thus integrates metacommunity models, which describe ecosystems as networks of networks, with large community evolution models. We find that rescue effects and source-sink effects occur within coupled habitats, which have the potential to change local selection pressures so that the local food web structure shows a fingerprint of its spatial conditions. Within our model system, we observe that habitat coupling increases the lifetimes of top predators and promotes local biodiversity.

## Introduction

All over the world, ecologist currently observe alarmingly high extinction rates, with spatial changes (such as habitat fragmentation or habitat loss due to land use) being one of the most important drivers^1, 2^. Biodiversity loss might entail a decreased level of ecosystem functioning^3^ and a decreased ability to provide essential ecosystem services^4, 5^. The exact consequences of such a biotic crisis are difficult to predict, but likely to persist for millions of years^6^. In order to develop reasonable conservation strategies before irreversible collapses take place, it is thus of utmost importance to understand how ecosystems respond to environmental changes and in particular how changes in the spatial topology influence local food web structure, stability and evolution.

Several authors highlight the need to study evolutionary metacommunities in this context of global change because the combination of adaptation, dispersal and species interactions can lead to unexpected, new phenomena (see refs 7–9, but more examples are given below). So far, much effort has been made to study metacommunities (for an introduction see ref. 10), but evolutionary aspects have often been neglected based on the controversal assumption that such processes take place on much longer time scales^11, 12^. The predictions derived from these non-evolutionary studies might nevertheless help to understand the enormous impact of the spatial context on local communities. For example, Gravel *et al*. studied complex food webs that were locally prone to extinctions, but whose complexity was observed to promote their persistence through regional dynamics^13^. Mougi and Kondoh showed that metacommunity complexity, measured by the number of local food webs and their connectedness, stabilizes food-web dynamics^14^. And Pillai *et al*. introduced a model that produced complex networks through spatial aggregation of very simple local networks^15^.

An explicit population dynamics approach was taken by Plitzko and Drossel^16^, who studied a system of coupled niche networks and found that intermediate migration rates enhance robustness. The explanation is based on spatial rescue effects, where species can re-invade habitats after becoming locally extinct, and on non-synchronous oscillations that can accomodate more species. The result is in line with the well-known study by Mouquet and Loreau, who predict that the local diversity shows a hump-shaped relation with dispersal strength^17^. However, the exact shape of the diversity-dispersal relationship turned out to be dependent on the spatial topology^16^. Also Holyoak found that the arrangement of patches affects predator persistence via rescue effects in microcosm experiments with two to four patches^18^. Richhardt *et al*.^19^ analysed this effect in more detail and found that the connectance and the centrality of the migration network topology best predict its impact on the local food webs. Regarding mutualistic networks instead of food webs, betweenness centrality was also reported to be a good predictor for local species number, interactions and nestedness, as shown for set of pollination networks in isolated hills of the Argentinian Pampas^20^.

The studies mentioned so far represent a wide range of different approaches to describe spatial processes and they also use different measures of biodiversity and stability. It is thus surprising that they all agree on one very important observation: The coupling of several communities can enhance local biodiversity and food web stability. It is however important to note that this is only true for idealized systems that neglect various spatial processes, such as the spreading of diseases or the invasion of fundamentally different alien species, which might lead to negative effects of habitat coupling on local diversity.

Unfortunately, none of the mentioned studies considers long-term processes, meaning that they all fail to provide convincing predictions for systems on eco-evolutionary time scales. To our knowledge, there are only few studies that consider evolutionary processes in addition to spatial processes and species interactions. A first example is the study by Loeuille and Leibold, who investigated a food web model with two plant and two consumer species on a patchy, heterogeneous environment^21^, where one of the plant species had evolving defence strategies. The authors demonstrate the emergence of morphs that could only exist in a metacommunity due to the presence of dispersal. In two other evolutionary metacommunity studies, the same authors analyzed settings with changing local conditions^22^ or with local negative feedback between the species and their environment^23^. Another example is the model by Ritterskamp *et al*.^24^, where species evolve in terms of body mass and in terms of an abstract trait axis that can be interpreted as space. The model shows a diverse set of different outcomes including static versus evolutionary dynamic trophic network structures in space. Two extreme outcomes have been predicted, when additionally considering climate change^8^: Resident species might adapt and thereby prevent colonization by non-resident species or species sorting might prevent an evolutionary response. Both outcomes have been observed in a spatially explicit eco-evolutionary model of competing species responding to climate warming^9^. However, Mooney and Cleland compiled a list of observed evolutionary consequences of the biotic rearrangements since the Age of Exploration^25^ and demonstrate that immigration of alien species as well as changes in the spatial environment can also be drivers of evolutionary processes.

Taken together, these studies indicate that the combination of spatial and evolutionary processes can lead to the emergence of new phenomena that can not be observed if only space or only evolution is considered. They reveal new mechanisms explaining species coexistence and point out that the long-term responses of ecosystems to environmental change might actually differ from predictions based on classical, non-evolutionary metacommunity models. However, the variety of results in this small number of studies indicates that the picture is far from being complete and that many questions are still left open. The purpose of our study is to contribute to closing this gap.

Our starting point are so-called evolutionary food web models or large community evolution models^26–28^. The network structure in these models is not static, but emerges in a self-organised manner from the interplay between (**a**) population dynamics and (**b**) the recurrent addition of new morphs, which either represents invasion or mutation events. Two well-known examples are the webworld model^29, 30^ and the matching model^31, 32^, which both describe species via vectors of many abstract traits. By contrast, Loeuille and Loreau introduced a model where body mass is the only evolving trait^33^, which results in large, multi-trophic communities that are easily comparable to empirical data. More dynamic and even more realistic networks emerge when considering three instead of only one trait, as done in the model by Allhoff *et al*.^34^ that we use here. The model allows for food web analysis on short (only ecological) and long (ecological+evolutionary) time scales and therefore provides new opportunities to study the impact of environmental change on network structure and stability. For example, Allhoff and Drossel showed that environmental changes will affect the overall biomass flow in a food web, leading to the opening or disappearance of viable niches and hence to a change in trophic structure and ecosystem functioning^35^.

Here, we extend the model by Allhoff *et al*.^34^ towards a spatial component by considering dispersal links between two or four habitats with different spatial topologies. A very similar approach, but based on the evolutionary food web model by Louille and Loreau^33^, has already been taken by Allhoff *et al*. in 2015^36^. The original, non-spatial model produces static network structures, which are determined by the model parameters and which do not show considerable species turnover after the initial build-up of the networks^33, 37^. The spatial model version accordingly confirms results that were already known from non-evolving studies, but it is of limited value for the analysis of changes in the evolutionary dynamics. The model that we use here already shows a much more diverse evolutionary behaviour in the original, non-spatial version. We therefore expect to observe a likewise diverse behaviour of the spatial version, revealing new results that arise from the interplay between space and evolution. If for example spatial rescue effects lead to higher persistence of certain species in the food web, then this effect will cascade into other parts of the network and change the selection pressure on other species. We therefore expect to find an overall change in the evolutionary species turnover and hence a fingerprint of the spatial context on the local food web structure that arises from the coupling (or de-coupling) of habitats. We will address theses issues by analysing the traits and life spans of successful mutants, as well as the structure and biomass distributions of the resulting networks. Our study aims at identifying evolutionary responses to habitat coupling. Other, non-evolutionary processes that might change the food web structure on short time scales, such as seasonal dynamics^38^ or spatiotemporal variation^39^ are not considered.

## The Model

We consider an evolutionary food web model (for reviews see refs 26–28). Evolutionary processes describe the emergence of new morphs, which are modelled as modifications of existing ones. Ecological processes (such as predation and competition) then determine the population sizes, the biomass flows, and whether the new morph population is viable in the environment created by the other morphs. The networks thus evolve in a dynamical, self-organised manner. We use the evolutionary food web model introduced by Allhoff *et al*.^34, 35^ to describe local communities, but we extend their approach by taking diffusive migration between several of such local communities into account. Our model hence describes food webs in spatially discrete, patchy environments. A morph in our model is able to (1) interact locally with others, (2) mutate according to our evolutionary rules and (3) migrate to neighbouring habitats in a metacommunity.

### Local population dynamics

**Figure 1:**
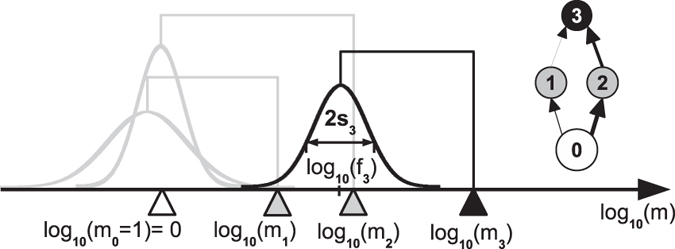
Model illustration using 4 morphs. Morph 3 (black triangle) is characterised by its body mass *m*_3_, the centre of its feeding range *f*_3_, and the width of its feeding range *s*_3_. The Gaussian function (black curve) describes its attack rate kernel *N*_3*j*_ on potential prey. Here, morph 3 feeds on morph 2 and 1 (grey triangles) with a high resp. low attack rate. Morph 1 and 2 are consumers of the external resource (white triangle). Also illustrated is the corresponding network graph. After^34^.

A morph *i* in our model is characterised by its average adult body mass, *m*_*i*_, the centre of its feeding range, *f*_*i*_, and the width of its feeding range, *s*_*i*_. These traits determine the feeding and competition interactions in the community and thereby the population dynamics, as illustrated in Fig. 1. A population of a morph *i* is represented by its biomass density *B*_*i*_. We follow the multi-species generalisation of the bioenergetics approach by Yodzis and Innes^40, 41^ in order to describe changes in biomass densities:

1 $${\dot{B}}_{0}={G}_{0}{B}_{0}-\sum _{j={\rm{consumers}}}{g}_{j0}{B}_{j}$$

for the external resource (with body mass *m*_0_ = 1) and

2 $${\dot{B}}_{i}=\sum _{j={\rm{resources}}}\,{e}_{j}{g}_{ij}{B}_{i}-\sum _{j={\rm{consumers}}}\,{g}_{ji}{B}_{j}\,-{x}_{i}{B}_{i}$$

for the consumer morphs. The coefficient *G*_0_ = *R*(1 − *B*_0_/*K*) describes the logistic growth of the external resource, with a growth rate *R* and a carrying capacity *K* = 100. The time scale of the whole system is defined by setting *R* = 1. The efficiency *e*_*j*_ is prey dependent and equals either 0.45 for feeding on the resource or 0.85 for preying on other morphs. *g*_*ij*_ is the mass-specific rate with which morph *i* consumes morph *j*, and *x*_*i*_ = 0.314 · *m*_*i*_^−0.25^ is a combined, mass-specific rate that describes *i*’s losses due to respiration and mortality. The parameter values are chosen as in refs 34 and 35 and based on the work by Yodzis and Innes^40^, Brown *et al*.^42^ and Brose *et al*.^41^.

The consumption rate is described via a Beddington-DeAngelis functional response^43^,

3 $${g}_{ij}=\frac{1}{{m}_{i}}\frac{{a}_{ij}{B}_{j}}{1+\sum _{k={\rm{res}}{\rm{.}}}{h}_{i}{a}_{ik}{B}_{k}+\sum _{l={\rm{comp}}{\rm{.}}}{c}_{il}{B}_{l}}\,,$$

where *a*_*ij*_ denotes the per capita rate of successful attacks of predator *i* on prey *j*, *a*_*ij*_. It is based on the Gaussian feeding kernel *N*_*ij*_, which is also illustrated in Fig. 1:

4 $${a}_{ij}={m}_{i}^{0.75}\cdot {N}_{ij}$$

5 $$=\,{m}_{i}^{0.75}\cdot \frac{1}{{s}_{i}\sqrt{2\pi }}\cdot \exp [-\frac{{({{\rm{l}}{\rm{o}}{\rm{g}}}_{10}{f}_{i}-{{\rm{l}}{\rm{o}}{\rm{g}}}_{10}{m}_{j})}^{2}}{2{s}_{i}^{2}}]\,\mathrm{.}$$

The parameter *h*_*i*_ = 0.398 · *m*_*i*_^−0.75^ in eq. (3) is the handling time of morph *i* for one unit of prey biomass, and *c*_*il*_ quantifies interference competition among predators *i* and *l*. It depends on their similarity, as measured by the overlap ${I}_{il}=\int {N}_{ij}\cdot {N}_{lj}d({{\rm{l}}{\rm{o}}{\rm{g}}}_{10}{m}_{j})$ of their feeding kernels, via

6 $${c}_{il}={c}_{{\rm{food}}}\cdot \frac{{I}_{il}}{{I}_{ii}}\,{\rm{for}}\,i\ne l\,\mathrm{.}$$

We assume that interference competition is higher within a morph population than between different morphs, e.g. due to territorial or mating behaviour^44^. We therefore introduce an intra-specific competition parameter *c*_intra_ and set *c*_*ii*_ = *c*_food_ + *c*_intra_. The influence of these competition parameters has been discussed in a previous article^34^: Stronger inter-specific competition widens the niche taken up by a given morph and thus results in fewer morphs, whereas stronger intra-specific competition has the opposite effect. It leads to smaller population sizes, reducing competition, so that more morphs can find a niche in the food web. Here we use two fixed parameter sets: *c*_food_ = 1.2/*c*_intra_ = 0.4 and *c*_food_ = 2.0/*c*_intra_ = 0.1, resulting in (isolated) food webs of approximately 27.2 ± 5.7 resp. 7.1 ± 1.6 morphs.

### Network evolution

Each simulation starts with a single ancestor morph with body mass *m*_1_ = 100 and feeding traits *f*_1_ = 1 and *s*_1_ = 1. The initial biomass densities are *B*_0_ = *K* = 100 for the resource and *B*_1_ = *m*_1_ · *ε* = 2 · 10^−4^ for the ancestor morph. The parameter *ε* = 2 · 10^−6^ is the local extinction threshold, i.e. the critical individual density required for a morph population to survive. A “mutation event” occurs every 10^4^ time units. Then, those morphs that went globally extinct (meaning that their population sizes on the different habitats all dropped below the local extinction threshold) are removed from the system. After that, one of the remaining morphs (but not the external resource) is chosen randomly as “parent” *i* for a “mutant” *j*. The logarithm of the mutant’s body mass, ${{\rm{l}}{\rm{o}}{\rm{g}}}_{10}({m}_{j})$, is chosen randomly from the interval $[{{\rm{l}}{\rm{o}}{\rm{g}}}_{10}(\frac{1}{2}{m}_{i}),{{\rm{l}}{\rm{o}}{\rm{g}}}_{10}\mathrm{(2}{m}_{i})]$, meaning that the body masses of parent and mutant differ at most by a factor of 2. The mutant’s initial biomass density is set to *Bj* = *m*_*j*_ · *ε* and taken from the parent. In some cases, this may put the parent below its extinction threshold. The mutation fails, if the parent biomass falls below zero. The logarithm of the mutant’s feeding centre, ${{\rm{l}}{\rm{o}}{\rm{g}}}_{10}{f}_{j}$, is drawn randomly from the interval $[({{\rm{l}}{\rm{o}}{\rm{g}}}_{10}({m}_{j})-3.5),({{\rm{l}}{\rm{o}}{\rm{g}}}_{10}({m}_{j})-0.5)]$, meaning that the preferred prey body mass is 3 to 1000 times smaller than the consumer’s body mass, consistent with the results from Brose *et al*.^45^. The width of the feeding range, *s*_*j*_, is drawn randomly from the interval [0.5, 1.5]. Several variations of these rules, including different mutation step sizes and direct inheritance of feeding preferences, have only minor impacts on the resulting network structures, as discussed in a previous article^34^.

### Migration between habitats

We chose a simple diffusion approach to describe migration between two (or more) equivalent habitats. In principle, each morph can have N populations in a metacommunity consisting of N habitats, one on each habitat. We indicate this with an additional index: *B*_*i*_^(*k*)^ describes the biomass density of morph *i* on habitat *k*, with *k* ∈ {1…*N*}. In order to describe the biomass flow between the habitats, we simply add the following migration terms to equation (2):

$$\frac{d{B}_{i}^{(k)}}{dt}=\ldots -{\mu }_{i}{B}_{i}^{(k)}+\sum _{k^{\prime} =neighbours}\frac{{\mu }_{i}}{{l}_{k^{\prime} }}{B}_{i}^{(k^{\prime} )},$$

where *l*_*k*_ is the number of links going out from the habitat *k*. We do not include loss terms, which means that all biomass leaving one habitat appears on the other(s). This approach is known as “patch-wise migration” from previous studies^16, 46^. Specific migration rates scale allometrically: *μ*_*i*_ = *μ* ⋅ *m*_*i*_^1/4^, with *μ* = 10^−4^. The resource is assumed to be confined to its habitat and does not migrate, *μ*_0_ = 0.

## Methods

We start our analysis with small metacommunities consisting of only 2 habitats (10 simulations for each scenario). In this case, we consider 4 different scenarios: (1) Migration sets in at the beginning of the simulation, so that both food webs co-evolve together. (2) Migration starts only after the initial build-up of the networks, mimicking situations in which separate ecosystems become coupled, e.g. via canals between water bodies or via the formation of land bridges. (3) Metacommunities arising from the first scenario become de-coupled and evolve further as isolated food webs. (4) Metacommunities arising from the second scenario become de-coupled and evolve further as isolated food webs.

After that, we extend our analysis to metacommunities with 4 habitats (5 simulations for each scenario). We focus on the ring and the star, as examples for spatial topologies with the same or differing number of links per habitat. Other topologies can be grouped into the same two categories and lead to qualitatively the same results, as summarized in the online supplementary material. Migration is either present during the whole simulation time (as in scenario 1) or it sets in after the initial build-up of the networks (as in scenario 2). For these more complex metacommunties, we had to decrease the total number of morphs (by adjusting the competition parameters) in order to keep the computational effort within reasonable boundaries.

In addition to the described spatial topologies, we also simulate food webs on isolated habitats, which are not coupled to others. This corresponds to the original, non-spatial model version^34, 35^) and serves as a reference. It allows us to clearly disentangle the effects arising from the spatial topology compared to those that also occur on isolated habitats.

For each of the described spatial topologies, and at each mutation event, we evaluate how many morphs and how much biomass is accumulated on each trophic level. We then average over time and over several simulations. Only data from fully emerged networks, but not from the initial network build-up is considered for our analysis. We also track the trait values and the flow-based trophic positions (after^47^) of all mutants, as well as the time spans that they are able to survive in the system. Those morphs that survive at least until the next mutation event are considered viable and counted in our analysis, while those mutants that go extinct directly after their emergence are neglected. Our data set thus allows us to study the impact of the spatial topology on the local trophic structure and on morph turnover rates.

We used the C language and numerical intergration (Runge-Kutta-Fehlberg method as implemented by GNU Scientific Library^48^) to perform our computer simulations. All simulations were run for 10^10^ model time units and principally produce as many networks as we perform mutation events. However, not all of these 10^6^ networks are independent replicates. A successful morph typically survives less than 10^4^ mutation events. Thus, after 10^4^ mutation events, we can expect to have a new network composition. This gives a rough estimate of approximately 100 independent replicates per simulation.

## Results

### Examplary simulation run

**Figure 2:**
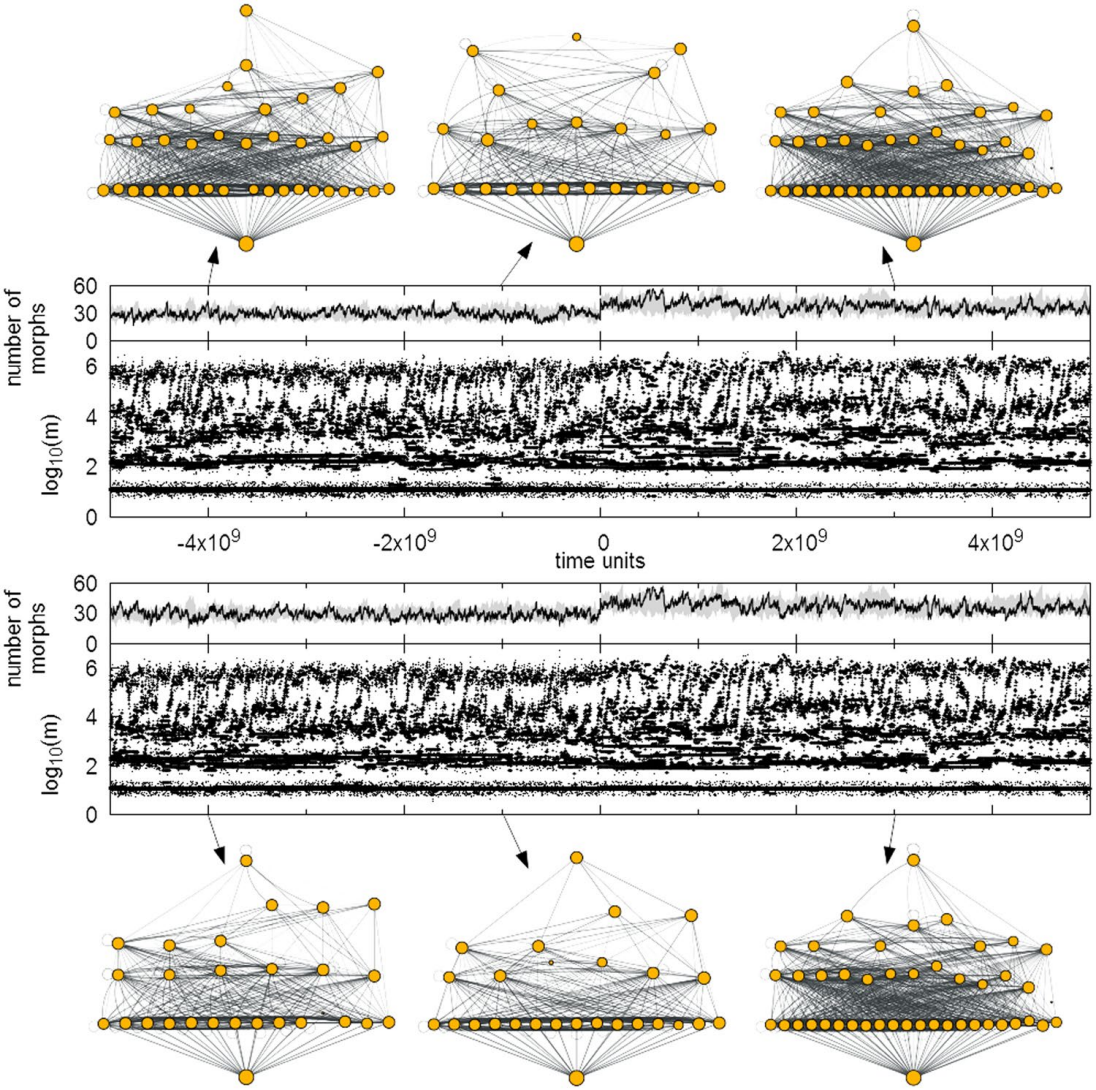
Cutout from a single simulation run for two habitats. Migration is switched on at time *t* = 0, according to scenario 2 (see methods section for more explanation). Each pair of panels shows respectively the local diversity (measured as the number of morphs present in that habitat) and the body masses of the morphs at a given time for one of two habitats respectively. Grey area shows morph number range for all 10 simulation runs. Network visualizations show examples of food webs at the marked time points. Resource is at the bottom, height shows trophic position and size of the nodes shows population sizes. Note that the food webs become identical after the coupling.

Figure 2 shows a typical simulation run for scenario 2. The two food webs initially evolve separately, but co-evolve together after the coupling. Thus, during the first half of the simulation, we observe the model behaviour as it is already known from previous studies^34, 35^. The morphs that emerge in our model have body masses that span several orders of magnitude and are concentrated in several clusters. The clustered structure reduces the predation pressure on a given prey, because its predators can feed on many alternative preys at the same time.

The resulting food webs are furthermore characterized by a continuous morph turnover, for which we assume two different mechanisms. The first driver of turnover has already been proposed by Allhoff *et al*.^34^. During food web evolution, predators tend to specialize on a single body mass cluster by adjusting their feeding center and by decreasing their feeding range, so that their total energy input is optimised. However, with the majority of predators concentrating on existing body mass clusters, new mutants can become viable outside of those. As such mutants will have few predation losses, they can easily out-compete other morphs, remove their feeding base, and cause extinction avalanches. (Note that this is fundamentally different to the well-known model by Loeuille and Loreau, where the body mass is the only evolving trait and where the feeding traits are fixed in relation to it, so that morphs outside of clusters don’t have a sufficient energy supply^33, 37^). More information on this first turnover mechanism is summarized in our online supplementary material.

The second turnover mechanism primarily affects top predators and is particularly relevant for our study. Some of the mutants can survive for a certain time span, even though their population densities remain very close to the extinction threshold. This is in particular true for morphs with large body masses on high trophic positions, since the biomass flow into high trophic levels is too weak to guarantee high growth rates. If such a morph mutates, there is a considerable chance that its biomass density drops below the threshold. A viable mutant can then easily replace its parent morph. But if the new mutant is not viable, then both parent and mutant become extinct, which resembles the so-called evolutionary suicide^49^. The loss of a predator then subsequently changes the viable niches in lower trophic levels, leading to evolutionary adjustments that cascade down the whole network.

The exemplary simulation run in Fig. 2 shows that coupled food webs show the same qualitative behaviour as isolated ones. We still observe the mentioned body mass clusters and a continuous turnover. However, we find that the food webs on the two habitats become identical and that their local diversity is considerably higher than the diversity of isolated habitats.

### Two coupled habitats

**Figure 3:**
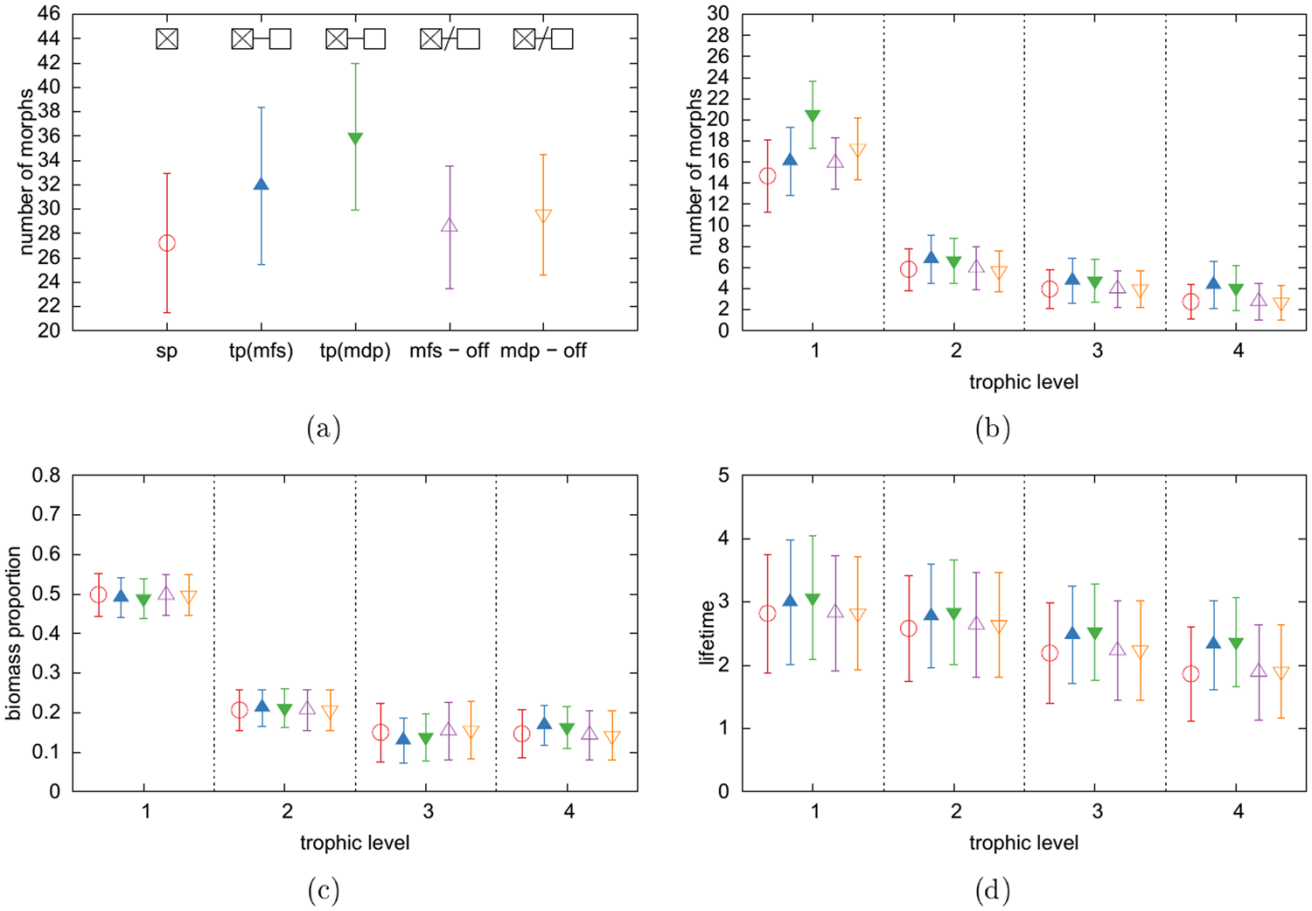
Properties of food webs of 5 different scenarios: single patch (sp), migration from the start (mfs), migration after a development phase (mdp) and migration switched off for the previous two scenarios. **(a)** Global diversity **(b)** Number of morphs per trophic level, as defined in Fig. 4(c) Distribution of biomass over trophic levels **(d)** Average lifetime, measured as decimal logarithm of the mutation events survived. Error bars correspond to standard deviations.

A broader analysis reveals that this result occurs in all of our simulations: The morph composition on both habitats becomes identical whenever the spatial coupling is active. Figure 3 summarizes data from isolated food webs and from food webs that were generated with the different scenarios presented in the methods sections. In panel a we show that the diversity is increased for coupled habitats (scenario 1 and 2) and surprisingly stays slightly increased after the habitats became decoupled (scenario 3 and 4). The food webs thus have a memory of the coupling that lasts at least as long as the duration of our simulations. Additional information on the network structure is then provided in panels b–d: We find that the increase in morph number is not restricted to a certain trophic level, but occurs in all of them. Moreover, we find that morphs in coupled habitats have in general a longer lifetime compared to morphs on isolated habitats. This is in particular true for the top trophic level, which is in line with the slightly increased biomass on this level and the slightly decreased biomass in the level below.

The increased lifetimes can be explained as follows: All viable morphs have two populations, one on each habitat. A parent’s population, which drops below the extinction threshold during a mutation process, can hence be rescued via re-immigration from the other habitat. The second turnover mechanism is thus absent as soon as migration is switched on. This primarily leads to a higher persistence of morphs in the top trophic level, which in turn leads to more persistent survival conditions in the trophic level below. It also keeps the populations in this level small, which decreases its total competition pressure, and hence allows for more morphs to emerge and exist there. The stabilizing features of the rescue effect for the top predators hence cascade down and lead to higher persistence and higher diversity on all trophic levels. However, the first turnover mechanism is still active, meaning that we still observe ongoing changes in the network structure.

In addition to these general results, we find a clear difference between scenario 1 and 2: Migration between pre-developed networks lead to an even higher diversity on the lowest trophic level than migration between co-evolved networks. Again, this can be interpreted as a memory effect, where some characteristics of the food web structure are still present long after the change in the spatial topology took place.

**Figure 4:**
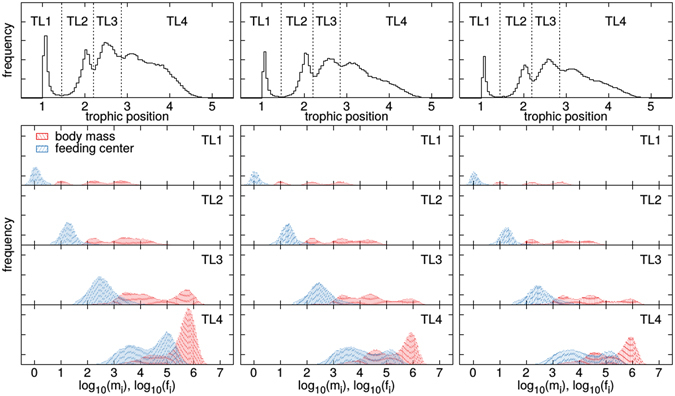
Properties of successful mutants: Top panels show the flow-based trophic positions of mutants in a single habitat (left panel), two habitats with migration from start (scenario 1, middle panel) and two habitats with migration after initial build-up (scenario 2, right panel). We use these distributions to group the morphs into 4 different trophic levels. The body masses and the feeding centers of the mutants in these levels are shown in the other panels. The data is normalized by the total number of mutants and includes only viable morphs.

For a deeper analysis, we studied the properties of successful mutants. Figure 4 shows the flow-based trophic positions, as well as the body masses and feeding centres of all mutants that survived at least until the next mutation event. Note that two morphs with identical feeding preferences and hence identical trophic positions can still differ in body mass. Trophic positions and body masses are thus only correlated, but there is no unambiguous assignment between them. Interestingly, Fig. 4 reveals no obvious differences between scenario 1 and 2, meaning that the history of the habitat coupling has no impact on morph turnover, although we have seen a memory effect in the network structure. However, we find once again a clear difference between isolated and coupled food webs: The number of successful mutations on trophic level 3 and 4 is lower in the coupled systems compared to the isolated system. The increased diversity in these levels does therefore not arise from a higher mutation success, but simply from extended lifetimes due to rescue effects.

### Four coupled habitats

**Figure 5:**
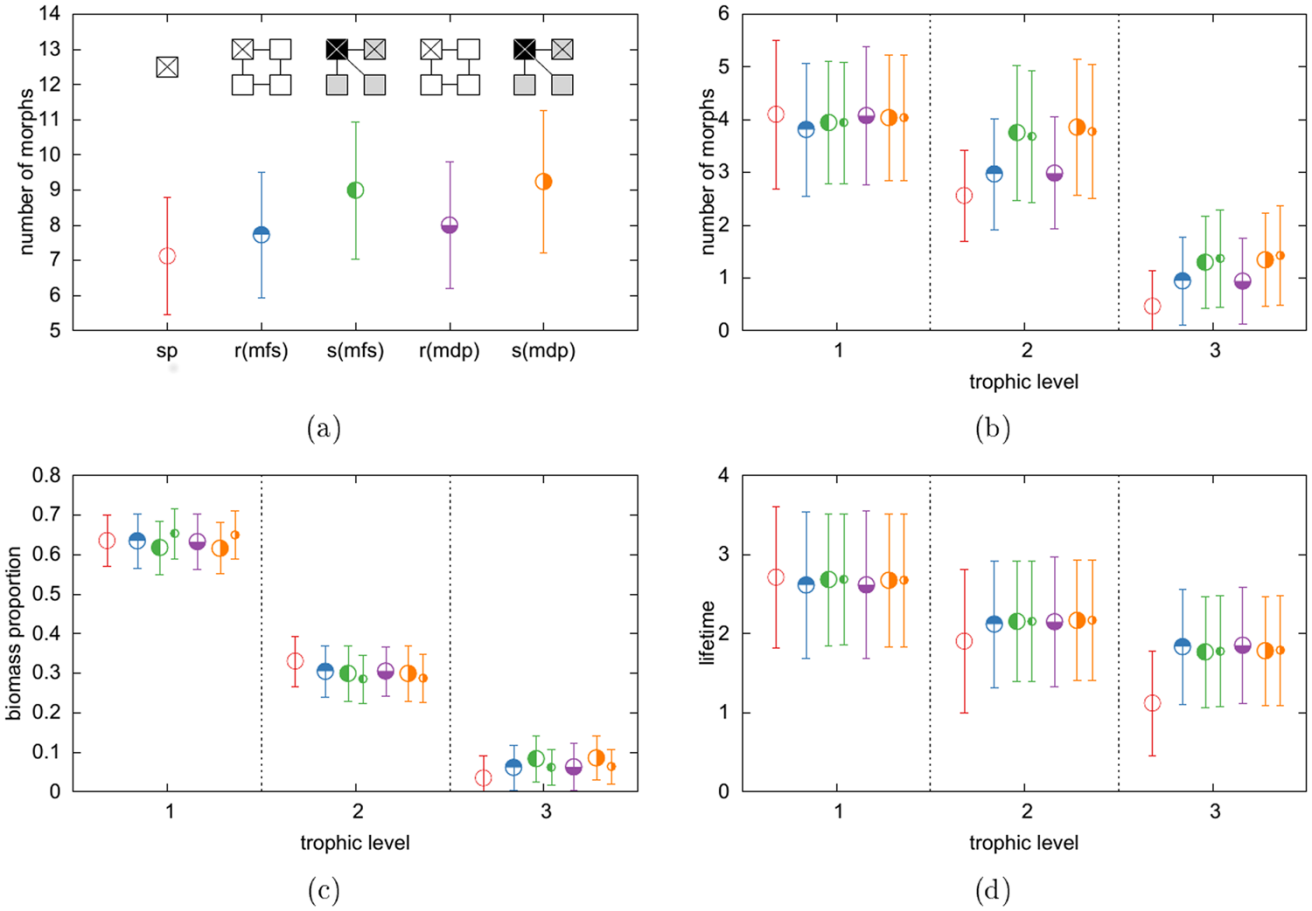
Properties of food webs of 5 different scenarios: single patch (sp), ring - migration from the start (mfs), star - migration from start (mfs), and migration after development (mdp) for the previous two scenarios. **(a)** Global diversity **(b)** Number of morphs per trophic level **(c)** Distribution of biomass over trophic levels **(d)** Average lifetime, measured as decimal logarithm of the mutation events survived. Data shown for the marked habitats, smaller symbols indicate the properties on the satelite patch. Error bars correspond to standard deviations.

As a final step, we analyse whether our results still hold in more complex spatial settings. We therefore repeat our analysis with topologies consisting of four instead of only two habitats. These simulations were performed with the second competition parameter set, which resulted in smaller food webs and hence shorter simulation times. However, the qualitative behaviour of the networks is not affected. Our results are summarized in Fig. 5. Additional information on four coupled habitats can be found in the online supplementary material.

The ring topology is an example of regular topologies (all habitats have the same number of links), which basically show the same results that we already know from the two habitat system: spatial coupling enables a rescue effect and hence leads to increased lifetimes of the morphs in high trophic levels. The effect cascades down to lower trophic levels and finally leads to an overall increased diversity.

The star topology on the other hand represents irregular topologies (the habitats differ in their number of links) and shows additional effects. The unequal number of links per habitat leads to an unequal biomass distribution as the central habitat receives all emigrating biomass from the three satellite habitats, but gives only one third of its emigrating biomass to each satellite. This results in a biomass increase on the central habitat and a biomass depletion on the satellite habitats. Morphs with large body masses, which typically also have a high trophic position, have a higher migration rate than morphs with small body masses, according to our choice of the allometrically scaled migration rate. The biomass flow is thus strong in high and weak in low trophic levels, which changes the steepness of the trophic pyramid in both habitats: Lower level morphs on the satellite habitats experience less predation pressure and accumulate more biomass, while the same morphs suffer from increased predation pressure on the center habitat and accumulate less biomass (see panel c). The increased predation pressure leads to smaller population sizes, which in turn reduces the competition pressure and hence opens new niches, which explains the increased number of morphs compared to the regular topologies. Increasing the migration rate increases the described imbalance between satellite and center habitat, as shown in the online supplementary material.

## Discussion

Our results confirm our assumption that the structure and evolution of a local community is strongly affected by the spatial context in which the community is embedded. We distinguish between regular (all habitats have the same number of migration links) and irregular (some habitats have more links than others) spatial topologies. Food webs that are coupled in a regular topology simply become identical, both in terms of morph composition and biomass accumulation. The top predators, which are prone to local extinction, can then be rescued by populations from neighbouring habitats. Their increased persistence subsequently changes the selection pressure in the trophic levels below, resulting in space-dependent evolutionary dynamics that finally lead to increased local diversities. Irregular topologies give rise to additional effects: The coupled food webs differ in terms of biomass accumulation due to the asymmetric migration flow, and these source-sink dynamics again change the local selection pressure both in the hub and in the satellite habitat. We therefore observe an even higher local biodiversity in irregular compared to regular spatial topologies of our model food webs.

Despite the significant impact of the spatial topology on the local network structure, we find only a weak effect on its biomass distribution. We explain this surprising observation with a dampening of trophic cascades in complex food webs^50^, which has already been discussed for the original, non-spatial model version^35^. Different spatial topologies change the persistence of top predators and hence the top-down control in the system, but the overall amount of energy available to the network is nevertheless determined by the external resource and thus the same for all spatial topologies studied here. This is in line with Gruner *et al*., who observes that bottom-up forces determine the maximum productivity and range of possibilities in a terrestrial community, while top-down forces govern the details of realized growth and structure^51^.

Our study is restricted to simple migration networks with only two or four habitats, in order to keep the simulation run times within reasonable boundaries, and we choose to present only the ring and the star topology as examples. We assume that bigger metacommunities with a much higher number of habitats reveal a more diverse set of outcomes. A successful morph that appears on one habitat might not have enough time to colonize all other habitats in complex spatial topologies before the next mutation takes place, which probably leads to heterogeneous species compositions despite habitat coupling. However, we performed extensive robustness checks and found that our results at least hold for other migration rates and other spatial topologies of small metacommunities, as discussed in the online supplementary material.

It is important to notice that the underlying mechanisms by which the spatial structure enhances survival in our model are already well-known from previous, non-evolutionary studies. The rescue effect was for example first mentioned forty years ago by Brown and Kodric-Brown^52^. It has been found both in laboratory experiments (e.g. in coupled microcosms^18^) and in field research (e.g. in fragmented heathland relicts^53^) and is today a well-known concept in metacommunity theory^10^. Its positive effect on the robustness of local food webs in large metacommunities has recently been demonstrated by Plitzko *et al*.^16^. A similar situation occurs for the source-sink-dynamics that we observe in irregular spatial topologies. The positive effect of these dynamics on local diversity have been known for several decades now^17, 54, 55^.

However, these spatial processes have so far been analysed on purely ecological time scales, without taking adaptation or evolution into account. Here, we could show that both the rescue effect and the source-sink effect have the potential to change the evolutionary dynamics within a local community. Even if they mainly affect top predators, other morphs in our model networks are indirectly affected via predation and competition interactions. Spatial coupling thus leads to changing selection pressures and finally to space-dependent local network structures. After all, we reach a conclusion that is surprisingly similar to what is already known from the non-evolutionary metacommunities mentioned in the introduction^13–16, 18^: Habitat coupling promotes local food web complexity. Our conclusion is likewise consistent with the positive effect of corridors on species richness that has been reported for moss microecosystems^56–59^. Our result is nevertheless a novelty, since we could show that this positive relation between spatial complexity and local biodiversity is not only true on ecological, but also on eco-evolutionary time scales.

To our knowledge, such long-term consequences of changing spatial structures have been largely overlooked, both because the idea of interacting spatial and evolutionary processes is still relatively new and because measuring these interactions in nature is more than challenging. However, there exists compelling evidence that evolutionary processes are often quick enough to affect ecological processes^11, 60–62^ and even to create eco-evolutionary feedbacks^63^, suggesting that predictions derived from non-evolutionary (metacommunity-) models should be reassessed. We think that a thorough understanding of the interplay between space and evolution is of utmost importance to provide reliable long-term predictions of ecosystem responses to current habitat loss or fragmentation issues, which might transform a spacious habitat into patchy environments. We hope that our study serves as a valuable starting point that will inspire further investigations into the same direction.

## Electronic supplementary material

Supplementary Information
