# Supplementary material for: Spatial topologies affect local food web structure and diversity in evolutionary metacommunities
Source: Sci Rep. 2017 May 12;7:1818. doi: 10.1038/s41598-017-01921-y (PMC5431821; doi:10.1038/s41598-017-01921-y)
Supplement: Supplementary file 1 — Supplementary Information [file 41598_2017_1921_MOESM1_ESM.pdf]

Spatial topologies affect local food web structure and diversity in evolutionary metacommunities
# - **Supplementary material** -

Lev Bolchoun[1], Barbara Drossel[2], and Korinna Theresa Allhoff[3*]

[1]Institute of Condensed Matter Physics, Technische Universität Darmstadt, Germany. lev_bo@fkp.tu-darmstadt.de
[2]Institute of Condensed Matter Physics, Technische Universität Darmstadt, Germany. drossel@fkp.tu-darmstadt.de
[3]Institute of Ecology and Environmental Sciences, Université Pierre et Marie Curie, Paris, France. korinna.allhoff@upmc.fr

March 12, 2017

Contents

A More information on turnover mechanisms **2**

B Transient effects directly after the coupling **5**

C The influence of the migration rates on our results **7**

D The impact of spatial coupling on ecosystem stability **9**

E Additional information on the ring and star topology **10**

F Additional information on other spatial topologies **12**

A More information on turnover mechanisms

New morphs entering the food web community in our model can displace already existent ones so that the emerging networks show a continuous turnover with extinction avalanches being followed by phases of network growth. We assume two turnover mechanisms that are responsible for this turnover. The second mechanism is particularly important for our study and discussed in detail in our main article. Here, we would like to show some more details on the first mechanism in order to provide a better understanding of the basic model.

At the beginning of a simulation, there are only a few morphs in the food web and the structure is strongly changed by every new mutant that enters the system. In this situation, being a generalist predator with a rather broad feeding range is a good survival strategy. It allows to feed on a wide range of body masses and to recognize new mutants as potential prey. However, as the network grows, it is more and more profitable for predators to become more specialized on established prey. Specialists predators exert a higher competition pressure than generalists, and can therefore easily displace them. The network thus becomes more and more a community of highly specialized morphs, which makes the community vulnerable. As the specialist predators are all concentrated on already existing body mass clusters, new mutants outside of those clusters might experience very little predation pressure and can therefore be highly successful. This allows such mutants to out-compete existing morphs, hence potentially remove the feeding base of other predators and cause major extinction avalanches. After such an extinction avalanche, the network grows again and being a generalists might be beneficial as long as structural changes take place...

Top predators are particularly vulnerable in this respect, both because they are dependent on all lower trophic levels and because their biomass density is typically close to the extinction threshold. The described specialization-extinction-cycle is therefore most prominent for high trophic levels, as shown in Figure A.1. From this time series, we observe that as the number of morphs in the top trophic level (bottom panel group) spikes, the mean feeding range (bottom panel) decreases. The situation becomes even clearer in Figure A.2, where we show that there are indeed fewer generalists for higher morph numbers. Particularly high values of the average feeding ranges, indicating a more generalists community, occur only after extinction avalanches, when the diversity is lowest.

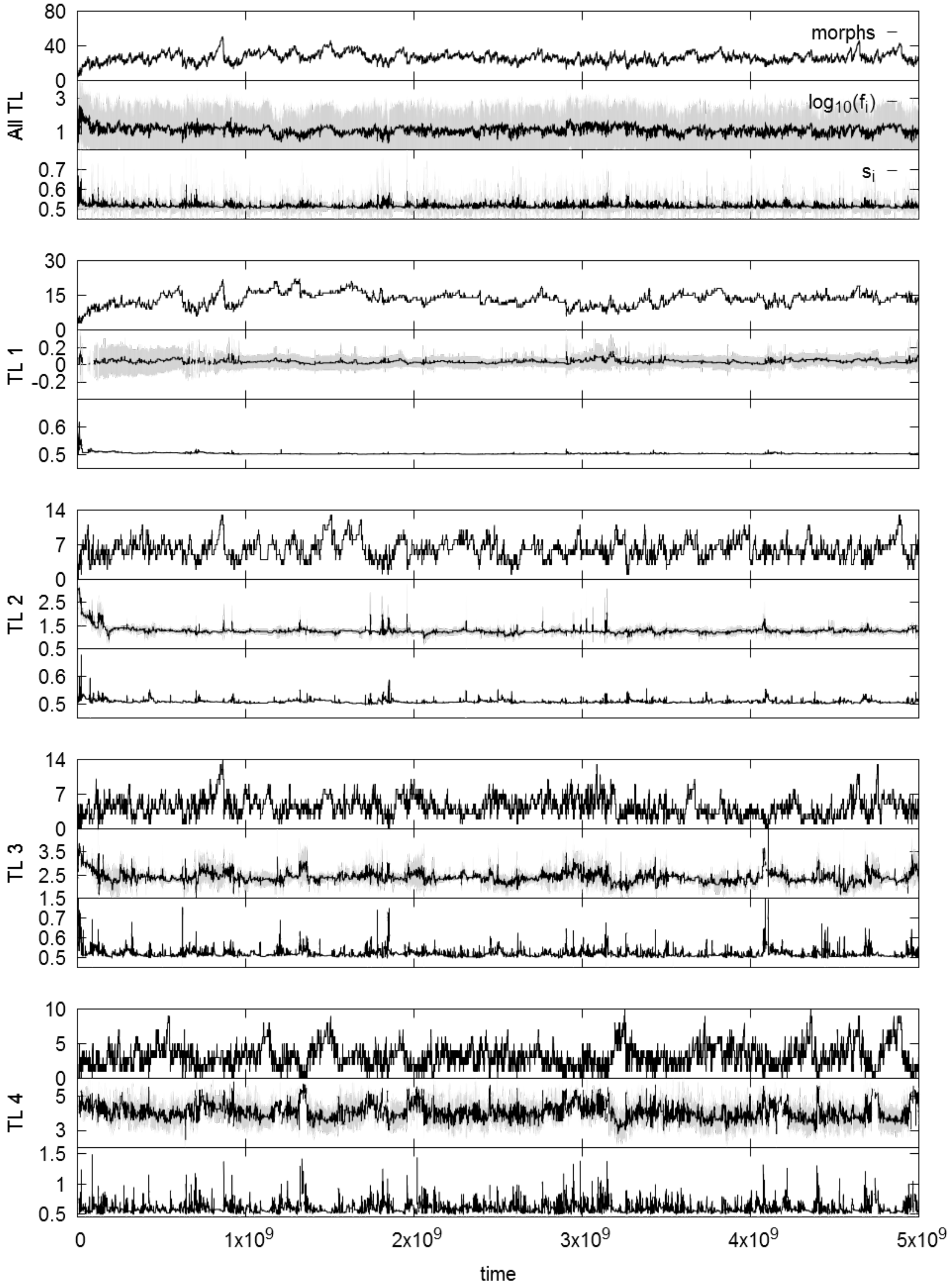

Figure A.1: Each group of three panels shows, respectively, from top to bottom: number of morphs, mean (black line) and standard deviation (gray area) of feeding center f_i, mean and standard deviation of feeding range s_i. The topmost group shows the whole food web, the other four groups show the data for each trophic level as defined in Figure 4 of the main article.

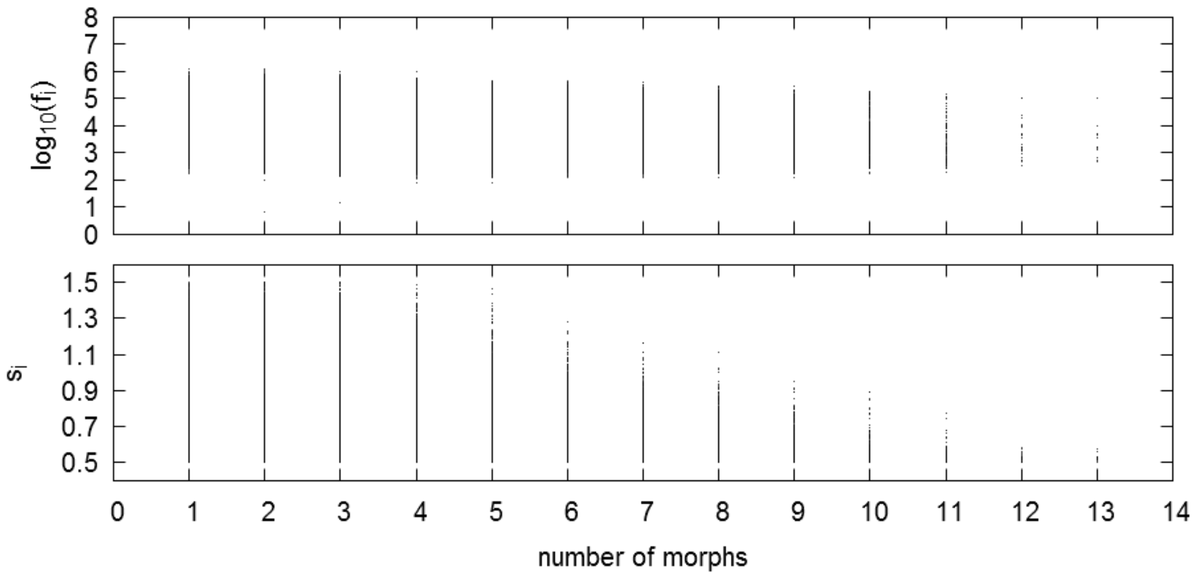

Figure A.2: Feeding center (top) and feeding range (bottom) of the morphs in TL 4. Data is taken from the timeseries shown in Figure A.1.

B Transient effects directly after the coupling

We are particularly interested in the long-term behaviour of coupled food webs and therefore mostly neglected all transient effects occurring directly after the coupling. However, we performed a number of additional simulations in order to clarify this point. For these simulations, we artificially switched the evolution off as soon as two habitats are coupled. This approach allows us to compare the short-term, purely ecological response with the long-term, eco-evolutionary response to habitat coupling.

Figure B.1 summarizes data from over 400 pairs of networks. Red circles indicate data from isolated habitats, as in the original model version introduced by Allhoff et al. in 2015. Blue squares indicate data from connected habitats, where evolution is switched off directly after the coupling. All morphs can thus migrate to the neighbouring habitat and start interacting with the native morphs. Not all, but most morphs survive this coupling and establish populations on both habitats, which explains the increased local diversity. After a while, the population dynamics simply reaches a fixed point, meaning that all population sizes converge to a constant value. Both food webs finally become identical and static. The blue error bars thus represent differences between simulation runs, but no fluctuations within single simulations. The coupling mainly affects the local food web diversity and the morphs composition (panel (a) and (b)), but the total biomass and the biomass distribution over the different trophic levels (panel (c) and (d)) stays approximately the same (see the discussion in the main article for an explanation).

The situation is completely different from the long-term scenario where evolution is not switched off (green triangles). The food webs show a continuous morphs turnover as a result of mutation and extinction events. The green error bars thus represent both fluctuations during single simulations and differences between simulation runs. The system directly after the coupling is more or less a superimposition of the two networks that became coupled, as explained above. We observe that not all morphs that could potentially survive in this non-evolutionary scenario are still viable in the eco-evolutionary scenario. New mutations disturb this "oversaturated" system, which leads to a decrease in food web diversity. However, the morph turnover mechanism is influenced by the coupling, so that the resulting number of morphs in coupled habitats is still higher than in isolated habitats, as explained in the main article.

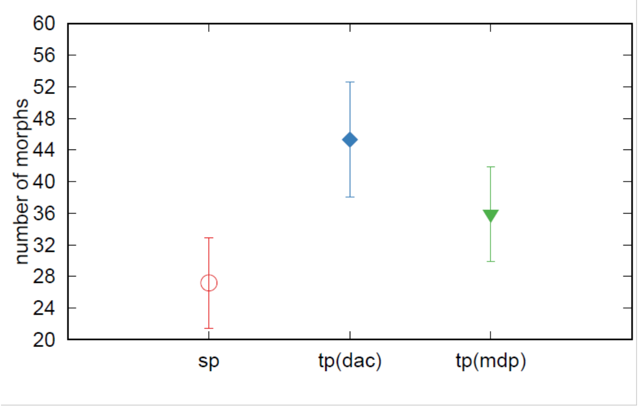

(a)

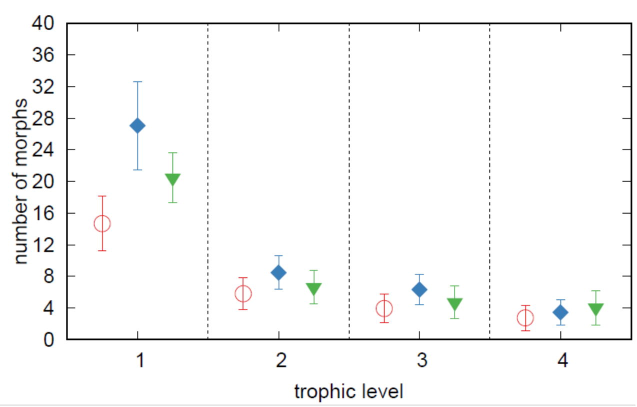

(b)

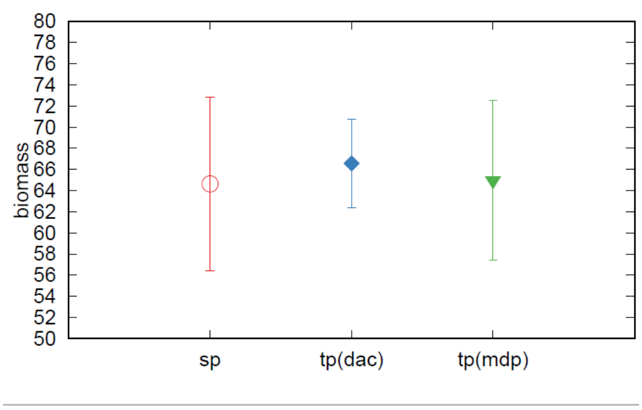

(c)

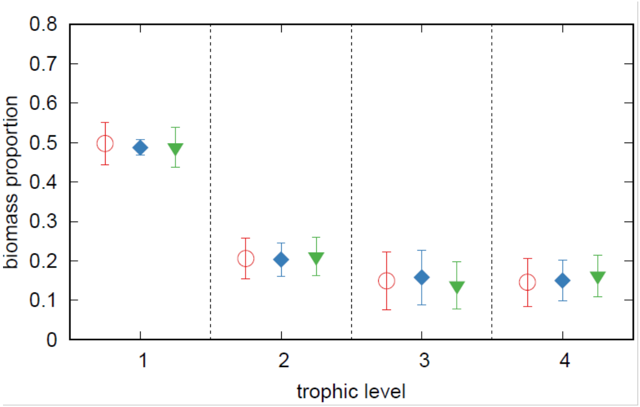

(d)

Figure B.1: Properties of isolated food webs (red circles), coupled habitats without evolution (blue squares), and coupled habitats with evolution (green triangles). **(a)** Local diversity. **(b)** Number of morphs per trophic level. **(c)** Total biomass density (without the resource). **(d)** Distribution of biomass over trophic levels .

C The influence of the migration rates on our results

We always observe identical networks on all habitats that are coupled in a regular topology. As emigration and immigration terms on each such habitat cancel each other out, the only result of migration is the rescue effect, regardless of the migration rate. The results of regular topologies are thus practically independent of the migration rate, as long as the migration rate is strong enough to homogenize the networks before the next mutation event takes place. The situation is slightly more complicated for irregular habitat configurations, because increasing migration rates increase the biomass imbalance between hub and satellite habitats. However, this does not affect the qualitative outcome, as shown in Figure C.1.

One main difference between our model here and the spatial version of the model by Loeuille and Loreau (Allhoff et al. "On the interplay of speciation and dispersal: An evolutionary food web model in space." Journal of theoretical biology 366 (2015): 46-56.) is the competition rule. This previous study uses a stand-alone quadratic competition term describing real biomass loss. Small migration rates can then lead to additional minipopulations of invasive morphs in the non-native habitat that are only maintained via immigration, but that are actually not viable because of strong competition with natives. Higher migration rates then lead to higher biomass gain from immigration, which eventually overcompensates these competition losses. Strong competitors are then able to replace local morphs and colonize all habitats, which finally leads to network homogenization. This transition from priority effects (See De Meester et al. "The Monopolization Hypothesis and the dispersal–gene flow paradox in aquatic organisms." Acta Oecologica 23.3 (2002): 121-135) towards a homogenization of coupled networks is in line with the idea of decreasing gamma-diversity with increasing migration rates, as described by Mouquet and Loreau: "Community patterns in source–sink metacommunities." The American Naturalist 162.5 (2003): 544-557.

However, we do not observe this transition in our present study, because we use a different way to describe interference competition. Competition is included into the functional response gij, instead of being described in a stand-alone term. Both versions co-exist in the literature and differ in the interpretation whether this loss term refers to time loss (the time spent with competition decreases the time available for predation) or to biomass loss (combat injuries or density dependent effects like disease spreading). The competition rule that we use in our present study allows for coexistence of competing morphs, because intraspecific competition is higher than interspecific competition. Better adapted invaders can therefore establish themselves and in some cases outcompete previously very abundant local morph populations. Small migration rates may decrease the speed of this process, but it will happen eventually. By contrast, the competition rule used in the previous study leads to priority effects of already established populations. Invaders are thus suppressed by natives - even though they might be better adapted.

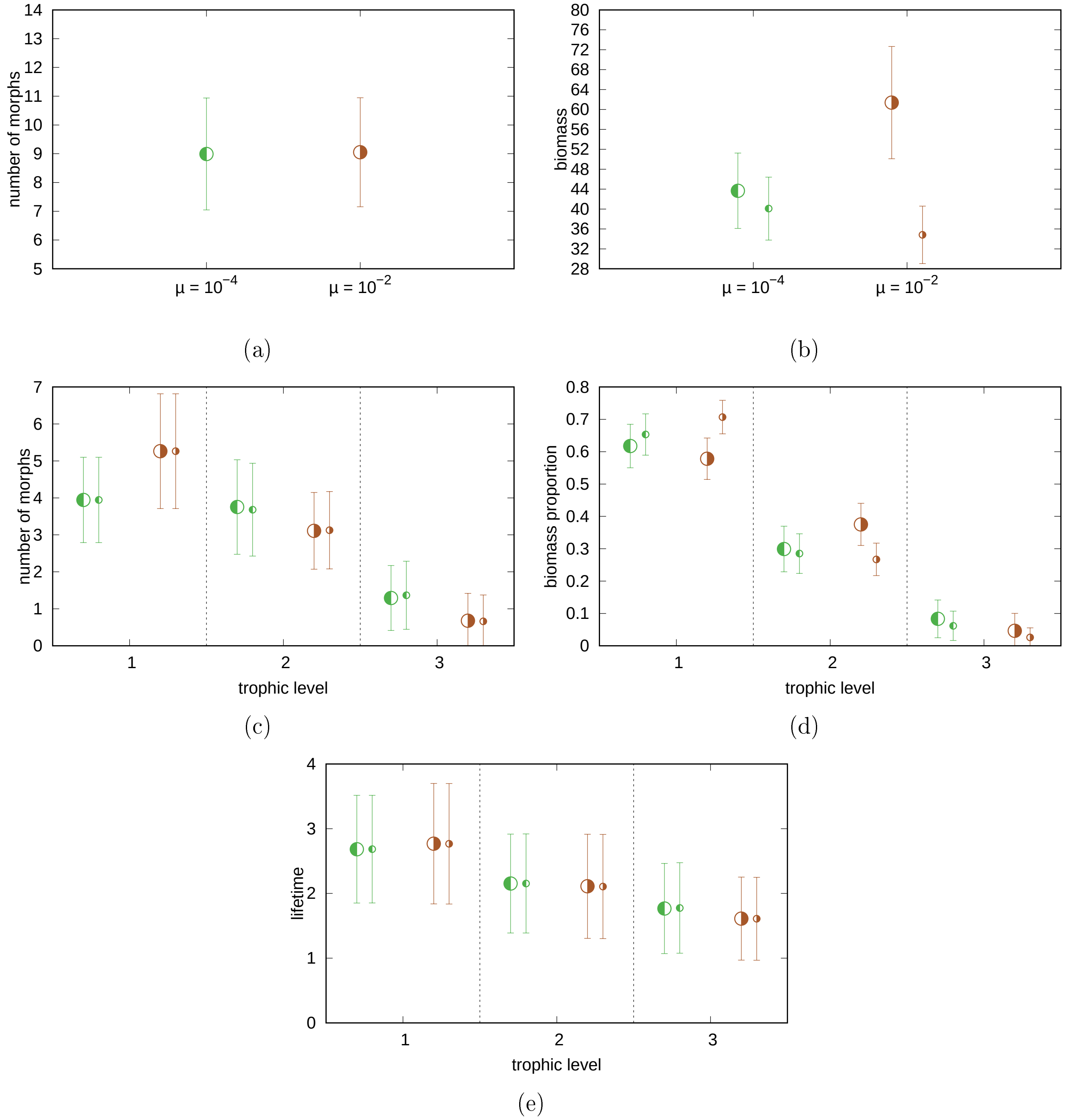

Figure C.1: Star configuration with the usual migration rate of $\mu = 10^{-4}$ (green) and with 100 times higher migration rate (brown). **(a)** Global diversity. **(b)** Total biomass density (without the resource). **(c)** Number of morphs per trophic level. **(d)** Distribution of biomass over trophic levels. **(e)** Average lifetime, measured as decimal logarithm of the mutation events survived.
For this figure, the TL were defined as in Figure E.2. Smaller symbols indicate the properties on the satellite habitat.

D The impact of spatial coupling on ecosystem stability

Not only the network structure, as discussed in our main article, but also the network stability is affected by the spatial context of the local community. A common measure for ecological stability is the relative size of biomass fluctuations and mean biomass, also known as the coefficient of variation. A low coefficient describes a rather stable situation with little variation, while a high coefficient describes high fluctuations over time. The same idea can also be applied to the fluctuations in the number of morphs as a measure for the evolutionary stability of the system. Both measures are evaluated in Fig. D.1 for two coupled (or decoupled) habitats. We observe that the spatial coupling of habitats generally increases both ecological and evolutionary stability. This is in particular true for the top trophic level.

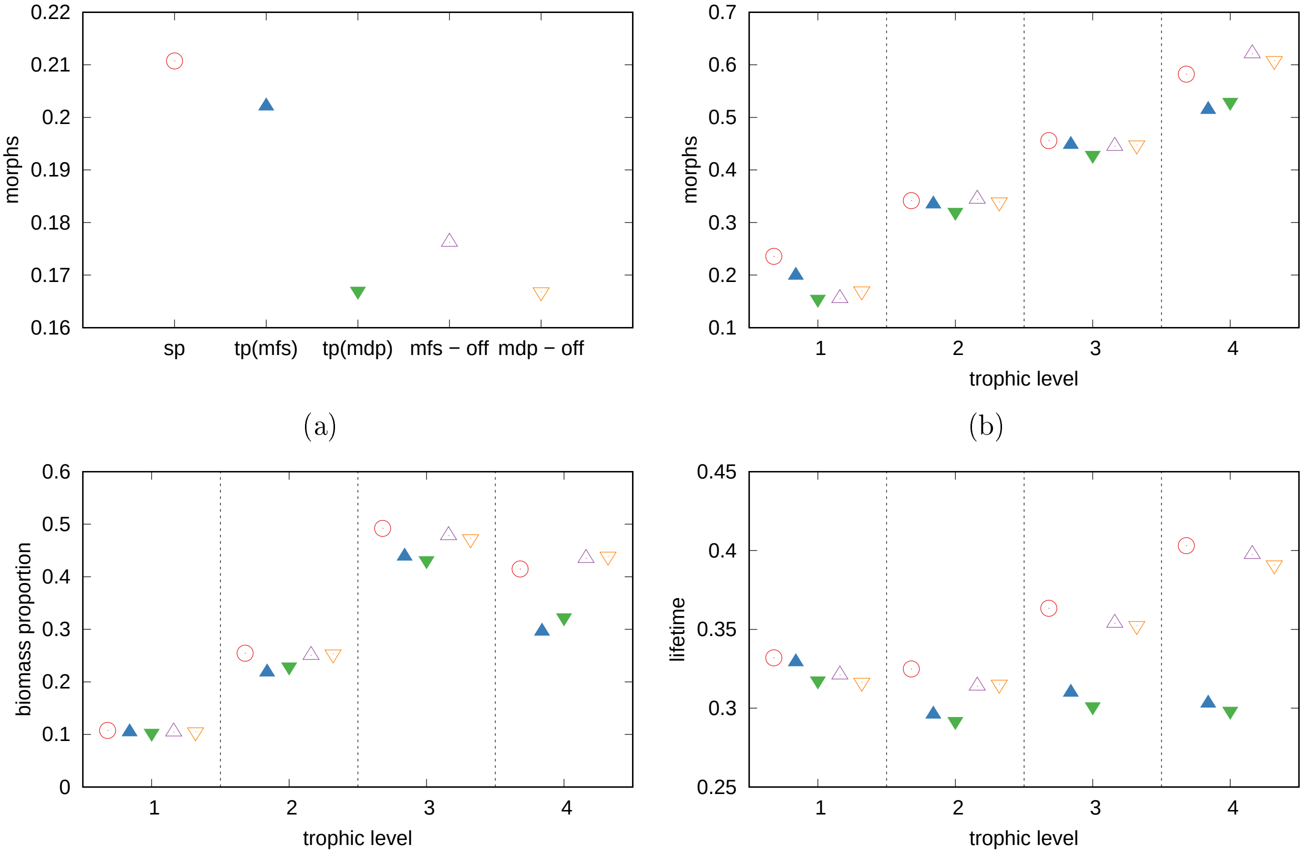

(c) (d)

Figure D.1: Coefficients of variation for several properties of single habitats (circles) and of two habitats with the four coupling scenarios explained in our main article (triangles). Competition parameters are $c_{\text{food}} = 1.2$ and $c_{\text{intra}} = 0.4$, as in Fig. 3 of the main article. **(a)** Global diversity **(b)** Number of morphs per trophic level **(c)** Distribution of biomass over trophic levels **(d)** Average lifetime, measured as decimal logarithm of the mutation events survived.

E Additional information on the ring and star topology

Our analysis of metacommunities with four habitats was analogous to the analysis of two coupled habitats. However, we decided to present only the two habitat system in detail in the main article in order to avoid redundancy. For the sake of completeness, we present here the missing Figures for larger metacommunities. Figure E.1 shows time series for food webs with competition parameters $c_{\text{food}} = 2.0$ and $c_{\text{intra}} = 0.1$ on different topologies and Figure E.2 shows how we grouped the different morphs into the distinct trophic levels that were used in Figure 5 of our main article.

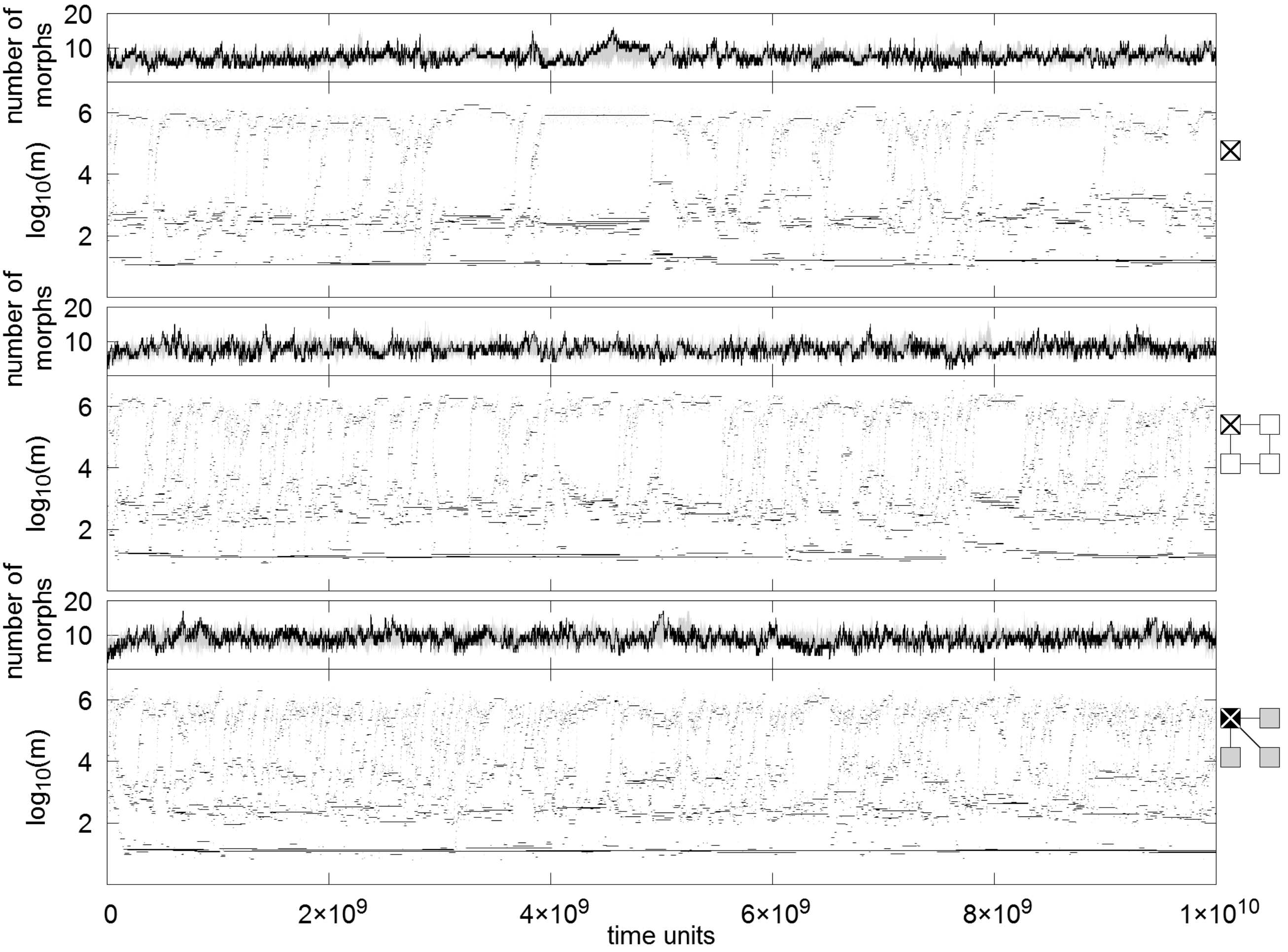

Figure E.1: From top to bottom: Time series for single habitat, ring, star. Data shown for the marked habitats.

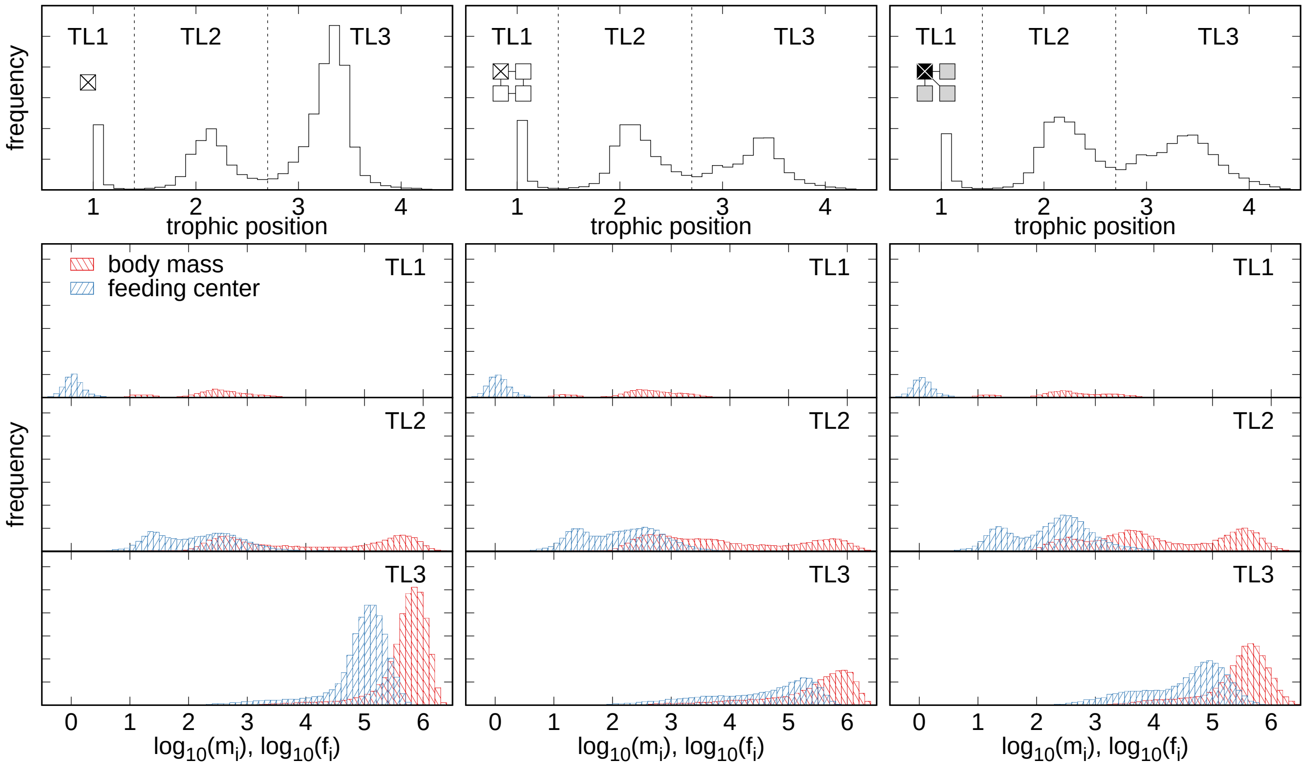

Figure E.2: Properties of successful mutants: Top panels show the flow-based trophic positions of mutants in a single habitat (left panel), ring topology (middle panel) or star topology (right panel). We use these distributions to group the morphs into 3 different trophic levels. The body masses and the feeding centers of the mutants in these levels are shown in the other panels. The data is normalized by the total number of mutants and includes only viable morphs.

F Additional information on other spatial topologies

In addition to the ring and star topology, which are discussed in detail in our main article, we also analysed other configurations of four coupled habitats. The results are summarized in the following three figures. Figure F.1 shows that all regular configurations generally produce the same behaviour as two coupled habitats: migration prevents the second turnover mechanism, stabilizes the top trophic level, which in turn keeps populations in the lower trophic levels smaller, reduces competition pressure and therefore allows for the emergence of more morphs.

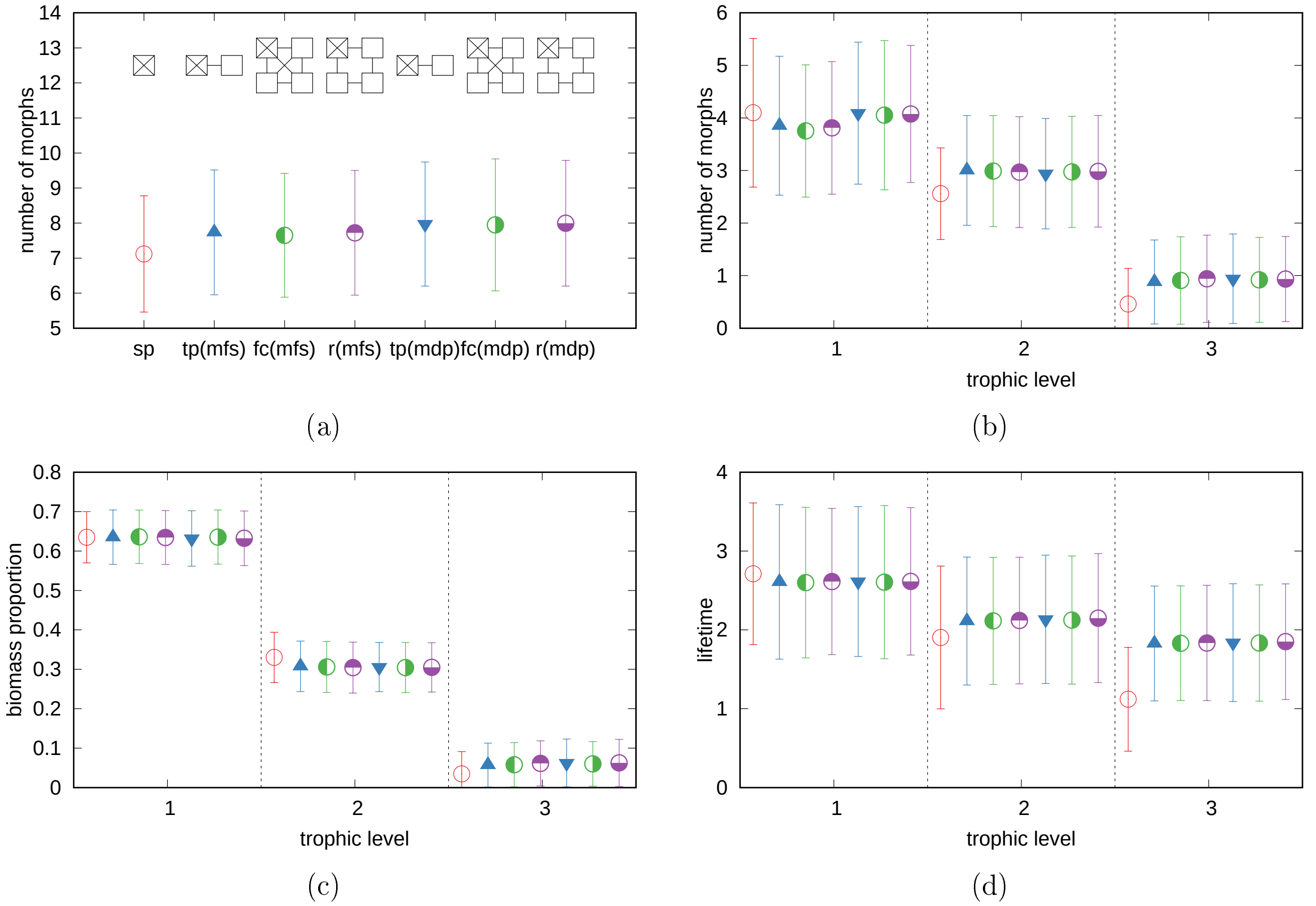

Figure F.1: Properties of food webs of 7 different configurations and scenarios: single habitat for reference, and 3 different regular configurations (two habitats, ring and fully connected four habitat configurations), each respectively with migration from the start and after a development phase. **(a)** Global diversity **(b)** Number of morphs per trophic level (as defined in Fig. E.2) **(c)** Distribution of biomass over trophic levels **(d)** Average lifetime, measured as decimal logarithm of the mutation events survived.

Figures F.2 and F.3 show the results for the four possible irregular configurations of four habitats. We assume that the results differ due to local differences between neighbouring nodes: because chain and diamond configurations have less imbalance between the rich and poor habitats the effects are weaker and diversity increase is correspondingly smaller than for star configurations. The mandolin configuration exhibits three different types of habitats, having three, two and one link respectively and therefore display the highest diversity.

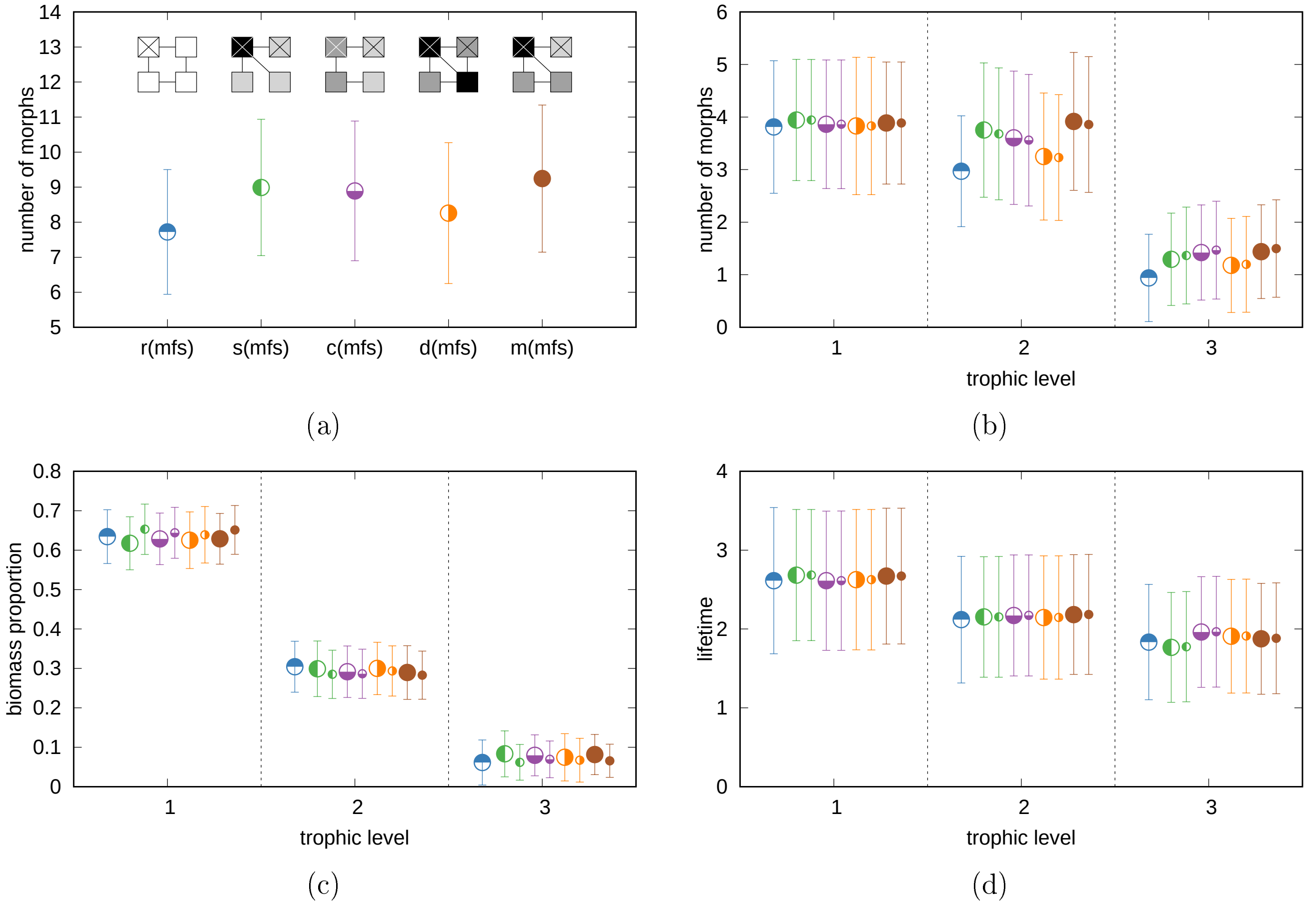

Figure F.2: Properties of food webs of 5 different configurations and scenarios: ring for reference, and 4 different irregular configurations (star, chain, diamond, mandoline), with migration from the start. **(a)** Global diversity **(b)** Number of morphs per trophic level (as defined in Fig. E.2) **(c)** Distribution of biomass over trophic levels **(d)** Average lifetime, measured as decimal logarithm of the mutation events survived.
Smaller symbols indicate the properties on the satellite habitat.

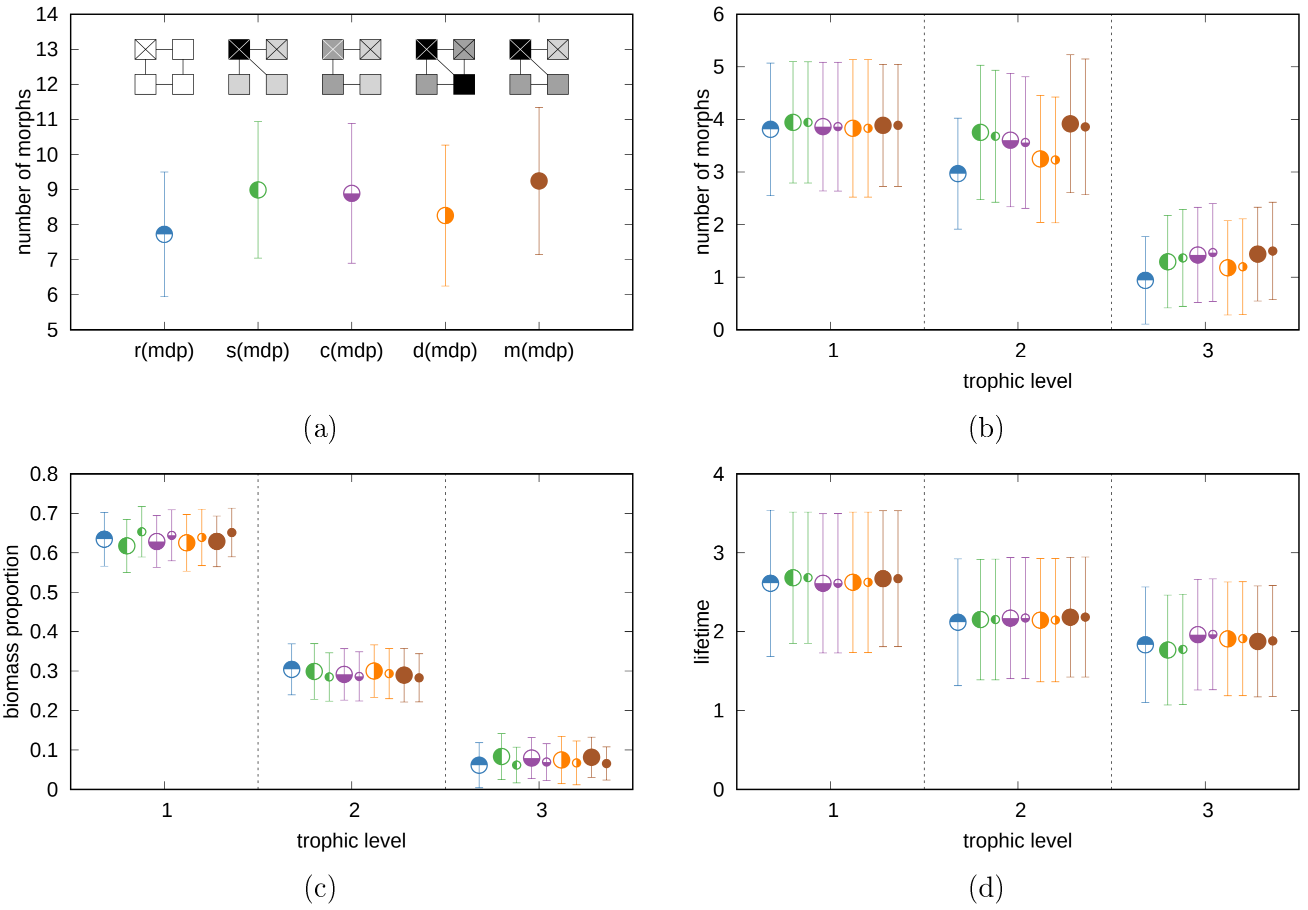

Figure F.3: Properties of food webs of 5 different configurations and scenarios: ring for reference, and 4 different irregular configurations (star, chain, diamond, mandoline), with migration on developed habitats. **(a)** Global diversity **(b)** Number of morphs per trophic level (as defined in Fig. E.2) **(c)** Distribution of biomass over trophic levels **(d)** Average lifetime, measured as decimal logarithm of the mutation events survived.
Smaller symbols indicate the properties on the satelite patch.
